# High-Performance Thin-Layer Chromatography-Densitometry-Tandem ESI-MS to Evaluate Phospholipid Content in Exosomes of Cancer Cells

**DOI:** 10.3390/ijms23031150

**Published:** 2022-01-21

**Authors:** María Sancho-Albero, Carmen Jarne, María Savirón, Pilar Martín-Duque, Luis Membrado, Vicente L. Cebolla, Jesús Santamaría

**Affiliations:** 1 Department of Chemical Engineering, Aragon Institute of Nanoscience (INA), University of Zaragoza, 50018 Zaragoza, Spain; msancho@unizar.es (M.S.-A.); jesus.santamaria@unizar.es (J.S.); 2 Networking Research Center on Bioengineering Biomaterials and Nanomedicine (CIBER-BBN), 28029 Madrid, Spain; mpmartind@gmail.com; 3 Instituto de Carboquímica, ICB-CSIC, 50018 Zaragoza, Spain; lmemgin@carbon.icb.csic.es; 4 CEQMA-CSIC, Facultad de Ciencias, Universidad de Zaragoza, 50009 Zaragoza, Spain; msaviron@unizar.es; 5 Instituto Aragonés de Ciencias de la Salud, IIS Aragón, 50009 Zaragoza, Spain; 6 Fundación Araid, 50018, Zaragoza, Spain

**Keywords:** HPTLC-MS, scanning densitometry, exosomes, phospholipids

## Abstract

The question of whether exosome lipids can be considered as potential cancer biomarkers faces our current limited knowledge of their composition. This is due to the difficulty in isolating pure exosomes, the variability of the biological sources from which they are extracted, and the uncertainty of the methods for lipid characterization. Here, we present a procedure to isolate exosomes and obtain a deep, repeatable, and rapid phospholipid (PL) composition of their lipid extracts, from embryonic murine fibroblasts (NIH-3T3 cell line) and none (B16-F1) and high (B16-F10) metastatic murine skin melanoma cells. The analytical method is based on High Performance Thin-Layer Chromatography with Ultraviolet and fluorescence densitometry and coupled to Electrospray (ESI)-tandem Mass Spectrometry (MS). Under the conditions described in this work, separation and determination of PL classes, (sphingomyelins, SM; phosphatidylcholines, PC; phosphatidylserines, PS; and phosphatidylethanolamines, PE) were achieved, expressed as µg PL/100 µg exosome protein, obtained by bicinchoninic acid assay (BCA). A detailed structural characterization of molecular species of each PL class was performed by simultaneous positive and negative ESI-MS and MS/MS directly from the chromatographic plate, thanks to an elution-based interface.

## 1. Introduction

Exosomes are usually defined as spherical extracellular nanovesicles from endocytic origin with a diameter between 20 and 150 nm, that are secreted to the extracellular media by almost all cell lines present in the organism. They transport a broad and complex range of active biomolecules, which are selectively and horizontally transferred to recipient cells from donor cells, evidencing the role of exosomes as intercellular communications vehicles both under physiological and pathological conditions [1].

Exosomes are formed by an aqueous inner space delimited by a lipid bilayer of ca. 5–10 nm-width, having an asymmetric distribution of lipid classes in the two leaflets of the bilayer. As several works pointed out, our current knowledge about composition and function of lipids in these vesicles is limited, mostly due to: the weakness of an exosome definition based on size and morphology; the variability of the biological sources from which they are extracted; and the uncertainty of methods for lipid characterization [2]. In this regard, uncertainties around 10% are usually considered acceptable in general lipidomic analysis using the gold standard electrospray (ESI)-Mass Spectrometry (MS) quantification [3], and uncertainties between 5% and 15% (or even greater) were obtained for most lipid species analyzed in exosomes by different methods combining chromatography and MS [2,4]. Errors mostly come from sample purity, lipid extraction, use of normalizers and standards, different ESI-MS responses of lipids affecting quantitation in determining a particular category, and other aspects derived from the analytical procedure itself.

Interestingly, it has been described that the composition of the lipid membrane of exosomes differs from that of the corresponding plasma membrane. Likewise, exosomes from different sources differ in their lipid composition, a fact which affects not only the stability of the exosomes, but also their participation in physiological and pathophysiological processes, such as cancer [5]. Thus, exosome lipids are currently being considered as potential biomarkers of some specific types of cancer [6,7,8]. 

Phospholipids (PL) account for 20–50% of all lipids in exosomes, with different percentages of their derived classes i.e., sphingomyelins (SM), phosphatidylcholines (PC), phosphatidylserines (PS), phosphatidylethanolamines (PE), phosphatidylinositols (PI), depending on the exosome source. PL from exosomes are important because their enzymatic degradation produces different classes of eicosanoids, which are involved in the development of cancer, among other diseases, promoting tumor growth by triggering cell proliferation, angiogenesis, and by decreasing apoptosis [9].

The PL composition of exosomes is not an exact representation of that in the parental cells, with some classes of them being depleted and other ones enriched in exosomes. For example, PC were depleted in exosomes derived from mesenchymal stem cells, U87 cancer cells, and Huh7 cells with regard to the parent cells. Similarly, PI were depleted from exosomes derived from all three cell lines, whereas PE were depleted from MSC but not from U87 exosomes. Likewise, although exosomes share a set of common lipid classes, their percentages of PL classes differed when exosomes from three different cancer cells were compared (SOJ6, PC-3 and U87) [5,7,10,11].

In the absence of specific molecular cancer markers, detailed characterization of exosomal PL classes, both quantitative (wt% or similar) and qualitative (identification of their molecular species), is of utmost importance to understand their role and/or establish a lipid signature of exosome malignant tumours with regard to their benign counterparts.

This work has the purpose of contributing to the characterization of PL of exosomes, and to study the variation of PL content in exosomes derived from three murine cell lines. For this, an original analytical procedure has been developed, which is based on exosome isolation and analysis of lipid exosome extract by High-Performance Thin-Layer Chromatography-densitometry directly coupled to MS.

Thin-Layer Chromatography (TLC) was used for lipid exosome analysis [2]. Although TLC has been a popular technique for separation of lipids, it has not usually been considered useful for neither an efficient separation nor a detailed characterization of a complex sample of lipids [12]. In this format, separated lipid bands were scraped off, followed by extraction from silica gel and subsequent filtration prior to MS analysis. This is a tedious, time-consuming procedure which gives poor recoveries and impractical when a large number of samples are processed. Recently, lipidome profiles of exosomes *vs* urinary microvesicles were obtained by scrapping off the separated sphingolipid and phospholipid bands which were qualitatively analyzed by Matrix-Assisted Laser Desorption/ionization (MALDI) [13] that has been the most used ionization mode coupled to HPTLC for lipid analysis [14].

The modern instrumental version of TLC, referred as to High-Performance Thin-Layer Chromatography (HPTLC), provides efficient separation of sample into lipid classes, detection and quantitative quantification by UV/FL densitometry, using the appropriate standards, and direct coupling with MS. In addition to MALDI, another approach to coupling is based on an on-plate automated elution-based interface which allows to locate the desired band, to extract it using an appropriate solvent and to transfer it to any MS instrument with Electrospray Ionization (ESI) [12], Atmospheric Pressure Chemical Ionization (APCI) [15], or other MS systems [16]. This makes it interesting for the analysis of lipids in exosomes along with other advantages such as: its speed of access to the selected hot bands on the plate, and the precision and representativeness of band extraction [17]. Likewise, ESI ion intensities are related to concentration of molecular species for a given separated lipid class [15]. Some examples found in recent literature illustrate this simple, rapid and powerful approach for structural determination of lipid molecular species of each class present in biological extracts, species such as ceramides [18], sphingolipids [15] or phospholipids [19] in lipidomic samples, by their *m*/*z*, and further confirmation by MS/MS data or other techniques. HPTLC separation contributes to the minimization of ion suppression problems, which emerge in MS-direct infusion techniques. Some particular characteristics of HPTLC are summarized in Table 1

Such a platform was used in this work for the first time for a rapid PL exosome determination. After isolating exosomes, we applied a rapid HPTLC-based method for separating main PL classes and for determining them by densitometry using the appropriate standards. Online in depth MS characterization of separated PL classes was performed directly from the chromatographic plate, providing ESI-MS profiles of their constituent molecular species. As requested in previous works [1,2], importance has been given here to the description of methodological details used to purify and isolate exosomes, and to analyze their extracted phospholipids, such that the studies can be reproduced. 

## 2. Results and Discussion

Conditions for HPTLC chromatographic development were targeted for separating most PL classes from each other and from other lipids in exosome extracts, and detecting and quantifying by densitometry. Separation was first monitored on silica gel plates by HPTLC migration distance (m.d., mm) using the standards mentioned in Experimental. The reason for using these particular standards is because each of them is the most abundant species in their corresponding PL class (SM, PC), as demonstrated below from the HPTLC-MS results.

The selected isocratic development in acidic medium allowed to separate at baseline most of standards corresponding to PL, over a total m.d. as short as 30 mm, as can be seen in Figure S1. On the other hand, phosphatidic acid (PA), hexosyl-ceramides (HexCer) and cholesterols (CHOL) migrated together the elution front at 30 mm. Structure of standards are also shown in Figure S1.

**Figure 1:**
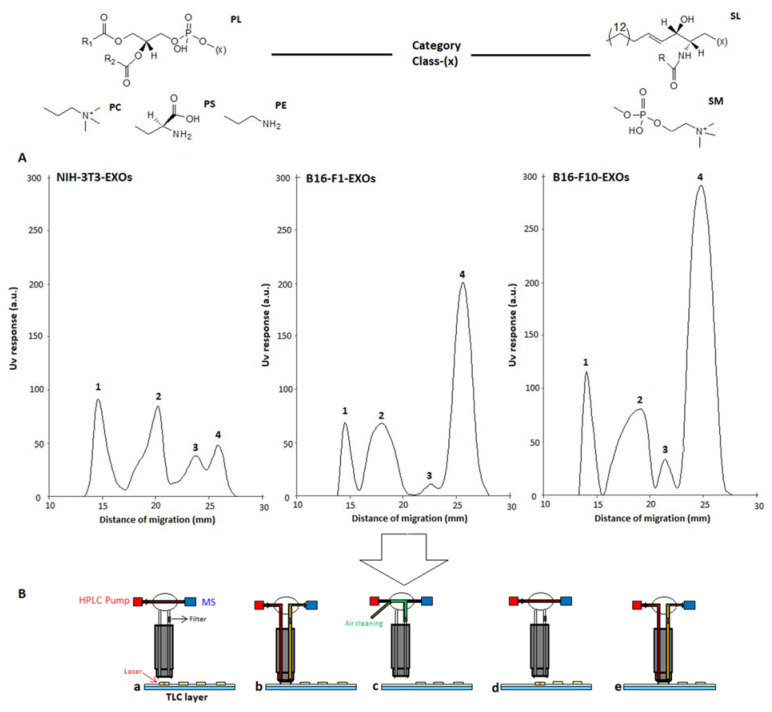
(**A**) HPTLC chromatograms (UV 190 nm) of exosomes lipid extracts showing separation of phospholipid classes (SM, PC, PS, PE). B16-F1-EXOs: peak 1: SM (m.d. = 14.6 mm); peak 2: PC (m.d. = 20.3mm); peak 3: PS (m.d. = 23.8 mm) and peak 4: PE (m.d.=25.9 mm). B16-F10-EXOs: peak 1: SM (m.d. = 14.5 mm); peak 2: PC (m.d. = 18.0 mm); peak 3: PS (m.d. = 22.6 mm) and peak 4: PE (m.d.=25.6 mm) NIH-3T3-EXOs: peak 1: SM (m.d. = 14.0 mm); peak 2: PC (m.d. = 19.1 mm); peak 3: PS (m.d. = 21.4 mm) and peak 4: PE (m.d. = 24.8 mm). (**B**) Automatic extraction of HPTLC bands using the TLC-MS elution-based interface. Red: HPLC pump for MeOH delivery (0.2 mL/min); blue: ion trap MS equipment; black: frit for silicagel filtering; +: laser crosshair. Idealized operation of peaks 1 and 2 extraction: (**a**) bypass; (**b**) first band extraction; (**c**) air cleaning; (**d**) bypass; (**e**) second band extraction.

Under these development conditions, SM, PC, PS and PE were properly separated from the studied exosome lipid extract samples as shown in Figure 1, where the corresponding HPTLC-UV chromatograms of NIH-3T3-EXOs, B16-F1-EXOs and B16F10-EXOs are shown, with a detail of m.d. of each PL class. In addition to comparing the migration distances of the standards and those of the separated bands in the samples, the assignment of PL classes and the structural identification of their molecular species were performed using HPTLC-ESI-MS profiling and MS/MS directly from the chromatographic plate using the described interface. Figure 1 also shows a detail of elution-based interface operation. The way of interface operating for extracting each peak is idealized there: (a) bypass position; (b) first band extraction position; (c) air pressurized cleaning position. The automated operation, which takes less than a minute to complete, is repeated for each extracted peak. Blanks of silica gel were extracted as control.

Intra- and inter-plate HPTLC repeatability results for the standards and PL classes of the samples are presented in Figure S2, including the Relative Standard Deviation (RSD)%, ±Area for 95% Confidence Interval (CI), calculation of the response factor (n = 3 × 3) and quantitative result for each PL class, expressed in µg/100 µg of exosome protein. Table 2 shows a summary of the quantitative results for the PL classes of each of the exosomes studied, determined by UV densitometry (190 nm).

### 2.1. Identity of PL Classes and Their Molecular Species

**Figure 2:**
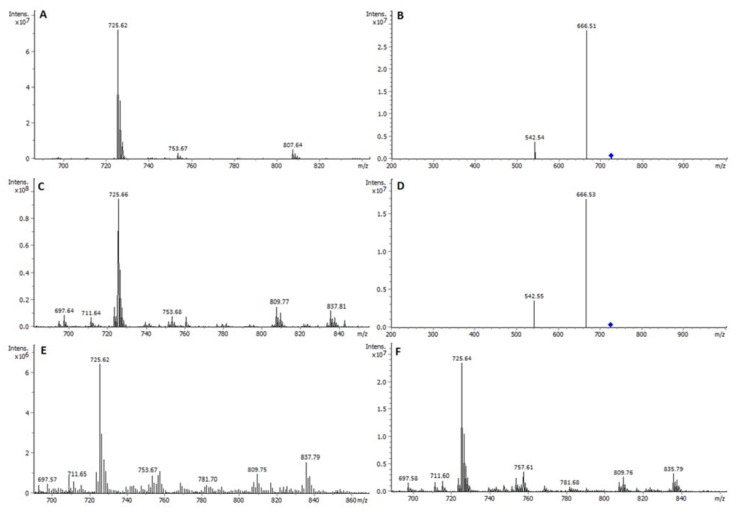
(**A**) HPTLC-ESI^+^-MS spectrum of SM standard. (**B**) HPTLC-ESI-MS/MS spectrum of the precursor ion at *m*/*z* 725.6 in the standard. (**C**) HPTLC-ESI^+^-MS spectrum of peak at 14.0 mm m.d. in NIH-3T3-EXOs sample. (**D**) HPTLC-ESI-MS/MS spectrum of the precursor ion at *m*/*z* 725.6, confirming peak to be SM in NIH-3T3-EXOs sample. (**E**) HPTLC-ESI^+^-MS spectrum of peak at 14.6 mm m.d. in B16-F1-EXOs sample. (**F**) HPTLC-ESI^+^-MS spectrum of peak at 14.5 mm m.d. in B16-F10-EXOs sample. For B16-F1 EXOs and B16-F10 EXOs, HPTLC-ESI-MS/MS spectra of the corresponding precursor ion at *m*/*z* 725.6 provides the same product ions than those of (**B**) and (**D**).

Assignment of PL classes and the molecular species found in them was performed by HPTLC-densitometry-ESI-MS spectra in positive (ESI^+^) and negative (ESI^−^) mode, and HPTLC-densitometry-ESI MS/MS. For each one of the studied exosomes, these spectra were obtained from several different lipid extracts on different plates, which proved to be repeatable. Obtained spectra from standards and PL classes in the studied samples are shown in Figures S3 and S4, respectively. Sphingomyelins (SM) were detected from the HPTLC-ESI^+^-MS spectrum of the band at the corresponding m.d. of each of the three lipid exosome extracts, shown in Figure 1. The most intense ion corresponded in all cases to the sodium adduct [C_39_H_79_N_2_O_6_P+Na]^+^ of d18:1; C16:0 SM (*m*/*z* 725.7). As the stability of [M+Na]^+^ was high, a consecutive fragmentation was achieved in the ion-trap MS. Hence, the respective HPTLC-ESI^+^-MS/MS spectrum of the precursor ion at *m*/*z* 725 [C_39_H_79_N_2_O_6_PNa]^+^ showed ion products at *m*/*z* 666.5 [M-N(CH_3_)_3_Na]^+^ and *m*/*z* 542.5 [M-phosphocholine+Na]^+^ (isolation width *m*/*z* 4 and amplitude voltage 0.4 V) (Figure 2). Likewise, the assignments were identical to the HPTLC-ESI^+^-MS/MS spectra of the SM standard substance. This confirms the sphingomyelin structure. 

Although ESI^+^ was the selected ionization mode for SM analysis, simultaneous ESI^−^ ionization of samples was an additional method to confirm structure identity. Therefore, an intense acetate ion [M+OAc]^−^ at *m*/*z* 761.9 was found which also confirmed **d18:1;C16:0** structure. Acetate ions come from acetic acid that is part of the mobile phase used for HPTLC separation. The following adducts of other SM molecular species were detected which were similar for the three studied extracts: d18:1;C14:0 (*m*/*z* 697.6 [C_37_H_75_N_2_O_6_P+Na]^+^; 733.8 [M+OAc]^−^), d18:1;C15:0 (*m*/*z* 711.6 [C_38_H_77_N_2_O_6_P+Na]^+^; 747.9 [M+OAc]^−^), d18:1;C18:0 (*m*/*z* 753.7 [C_41_H_83_N_2_O_6_P+Na]^+^; 789.9 [M+OAc]^−^), d18:1;C20:0 (*m*/*z* 781.7 [C_43_H_87_N_2_O_6_P+Na]^+^), d18:1;C22:0 (*m*/*z* 809.8 [C_45_H_91_N_2_O_6_P+Na]^+^), d18:1;C24:0 (*m*/*z* 837.9 [C_47_H_95_N_2_O_6_P+Na]^+^).

All spectra were obtained in duplicate in different days on different lipid extracts of each one of the studied exosomes (Figure S4).

Mass spectra show the majority presence of SM species with saturated fatty acyls in exosome extracts. Two monounsaturated species were detected at low concentrations, such as d18:1; C22:1 (*m*/*z* 843.7 [C_45_H_89_N_2_O_6_P+OAc]^−^ for B16F1 and B16F10), and d18:1;C24:1 (*m*/*z* 871.9 [C_47_H_93_N_2_O_6_P+OAc]^−^ for B16F10).

**Figure 3:**
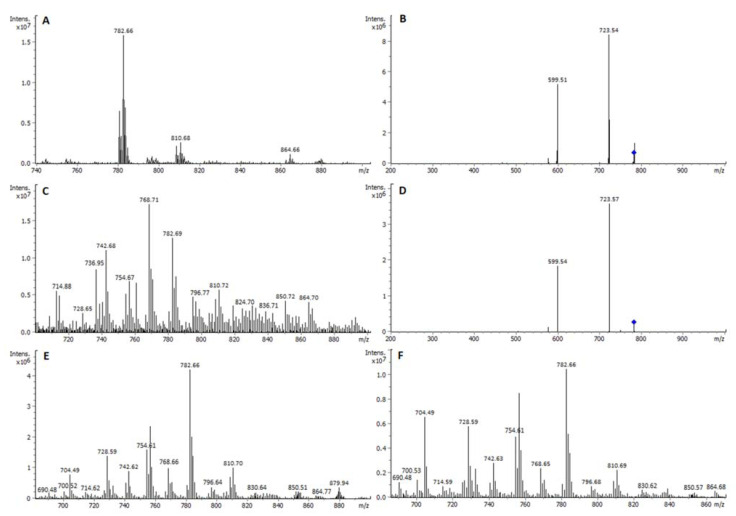
(**A**) HPTLC-ESI^+^-MS spectrum of PC standard. (**B**) HPTLC-ESI-MS/MS spectrum of the precursor ion at *m*/*z* 782.6 in the standard. (**C**) HPTLC-ESI^+^-MS spectrum of peak at 19.1 mm m.d. in NIH-3T3-EXOs sample. Most intense ion at *m*/*z* 768.7 (PC33:1). (**D**) HPTLC-ESI-MS/MS spectrum of the precursor ion at *m*/*z* 782.6 (PC34:1), confirming peak to be PC in NIH-3T3-EXOs sample. (**E**) HPTLC-ESI^+^-MS spectrum of peak at 20.3 mm m.d. in B16-F1-EXOs sample. (**F**) HPTLC-ESI^+^-MS spectrum of peak at 18.0 mm m.d. in B16-F10-EXOs sample.

Phosphatidylcholines (PC) were detected from the HPTLC-ESI^+^-MS spectrum of the band at m.d. specified in Figure 1. Simultaneous ESI^+^ and ESI^−^ ionization were performed. As in the case of SM, ESI^+^ was selected for HPTLC-ESI^+^-MS/MS spectra recording from the corresponding precursor ions (Figure 3).

HPTLC-ESI^+^-MS spectrum of the PC standard showed sodium adducts [C_42_H_82_O_8_PN+Na]^+^ at *m*/*z* 782.7, [C_44_H_86_O_8_PN+Na]^+^ at *m*/*z* 810.7, and [C_48_H_92_O_8_PN+Na]^+^ at *m*/*z* 864.7, which were assigned to a mixture of most abundant PC (34:1), and less abundant PC (36:1) and PC (40:2). As in the case of SM, these structures were confirmed by their corresponding [M+OAc]^−^ adducts at *m*/*z* 818.8, 834.7 and 900.7, respectively, by simultaneous ESI^−^ ionization. Likewise, HPTLC-ESI^+^-MS/MS confirmed these assignments: the precursor ion at *m*/*z* 782.7 was isolated and fragmented. The corresponding MS/MS spectrum displayed ion products at *m*/*z* 723.5 and 599.5 (isolation width *m*/*z* 4 and amplitude voltage 0.4 V), which corresponded to the loss of trimethylamine [–N(CH_3_)_3_] and phosphocholine [–(CH_2_)PO_4_N(CH_3_)_3_] groups, respectively. As in the case of SM, the most intense PC ion in the standards was also the most intense PC one in the studied lipid exosome extracts, which justifies the pertinence of the selection of the standards (Figure 3). This also happened in the case of the other separate classes, i.e., PS and PE, as shown below.

The most abundant ion in B16-F1 and B16-F10 exosome samples correspond to PC (34:1) species. Although this ion is also intense in the NIH-3T3 sample, the most abundant one in this non-malignant sample corresponds to PC (33:1) at m/z 768.7 [C_41_H_80_NO_8_P+Na]^+^; 804.8 [M+OAc]^−^ (Figure 3). Further research is needed to know whether this difference in PC composition may be used as a potencial biomarker linked to cell malignancy.

Similar ESI-MS profiles, ranging from 28 to 41 carbon atoms, including saturated, mono-, and di-unsaturated species, were obtained for the three lipid extracts of the exosomes studied. Other adducts of PC were also identified: PC (28:0) (*m*/*z* 700.5 [C_36_H_72_NO_8_P+Na]^+^), PC (29:0) (*m*/*z* 714.6 [C_37_H_74_NO_8_P+Na]^+^), PC (30:0) (*m*/*z* 728.6 [C_38_H_76_NO_8_P+Na]^+^; 764.9 [M+OAc]^−^), PC (31:0) (*m*/*z* 742.7 [C_39_H_78_NO_8_P+Na]^+^; 778.8 [M+AcO]^−^), PC (32:1) (*m*/*z* 754.7 [C_40_H_78_NO_8_P+Na]^+^; 790.9 [M+OAc]^−^), PC (34:1) (*m*/*z* 782.7 [C_42_82_5_NO_8_P+Na]^+^; 818.8 [M+OAc]^−^), PC (35:1) (*m*/*z* 796.8 [C_43_H_84_NO_8_P+Na]^+^), PC (36:1) (*m*/*z* 810.7 [C_44_H_86_NO_8_P+Na]^+^), 846.8 [M+OAc]^−^), PC (36:2) (*m*/*z* 830.7 [C_44_H_84_NO_8_P+2Na-H]^+^), PC (37:1) (*m*/*z* 824.7 [C_45_H_88_NO_8_P+Na]^+^), PC (38:2) (*m*/*z* 836.7 [C_46_H_88_NO_8_P+Na]^+^), PC (39:2) (*m*/*z* 850.7 [C_47_H_90_NO_8_P+Na]^+^), PC (40:2) (*m*/*z* 864.7 [C_48_H_92_NO_8_P+Na]^+^). A small number of PC species with six unsaturations were detected at low concentrations: PC (39:6) (*m*/*z* 878.8 [C_47_H_82_NO_8_P+OAc]^−^), and PC (40:6) (*m*/*z* 892.7 [C_48_H_84_NO_8_P+OAc]^−^).

**Figure 4:**
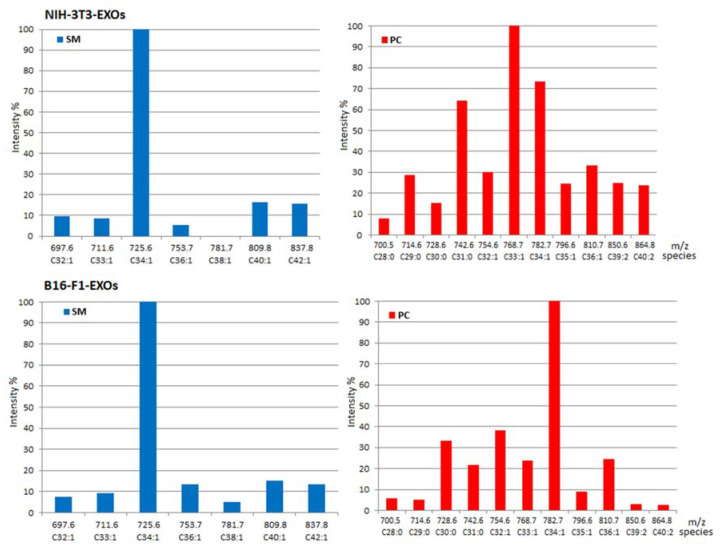

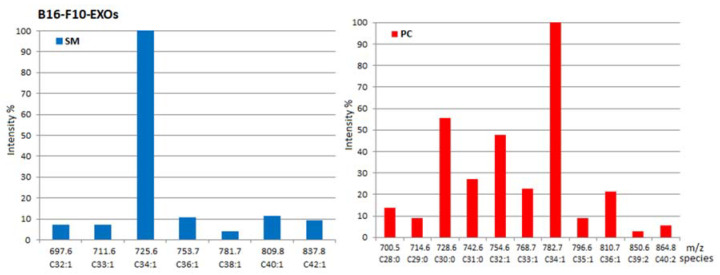
Normalized profiles of lipid species in the corresponding SM and PC classes of studied exosomes, obtained by HPTLC-densitometry-ESI^+^-MS (X-axis: *m*/*z* of ions and their corresponding species.

Figure 4 provides a summary of the species found in SM and PC classes of NIH-3T3-EXOs, B16F1-EXOs and B16F10-EXOs. In Figure 4 normalized ESI-ion intensities are depicted for each PL class separated (SM or PC) which are related to the corresponding concentration of species. Intensity-concentration relationship comes from three considerations. First, ESI-MS response per mass unit depends mostly on the head class group and, for a given class, the influence of fatty acyl chain length is far less significant [3]. Second, ESI ionization in HPTLC-densitometry-MS was carried out under the same conditions for each species. This should be explained: After HPTLC separation, the developed mobile phase is removed by drying before densitometry. Subsequently, each band is extracted by the interface using MeOH and the extract is sent to the mass spectrometer. As methanol is the common ionization solvent for all the bands, ionization is carried out under the same conditions for the separated PL classes. Therefore, no gradients were employed, avoiding gradient effect on nebulization and further individual lipid response.

Concerning PS, the species PS36:2 (at *m*/*z* 786.8, C_42_H_78_NO_10_P as [M-H]^−^) was identified by MS/MS in the lipid extract of exosomes, by the loss of the corresponding serine group (width *m*/*z* 4 and amplitude voltage 0.45 V) (Figure S4).

Regarding PE, species PE(36:2) at *m*/*z* 766.6 [C_41_H_78_NO_8_P+Na]^+^; 742.8 [C_41_H_78_NO_8_P]^−^ as [M-H]^−^ was found as the most abundant PE species in the samples which was confirmed by their MS/MS spectrum obtained using negative ESI mode (width *m*/*z* 4 and amplitude voltage 0.4 V). The ionization efficiency of PE species as protonated and/or sodium adducts is lower than that of other classes such as PC or SM, as the quaternary amine of PC with positive charge is much more stable than the primary amine of PE (Figure S4).

No species corresponding to PI or PA were identified. Several species of HexCer were detected in the front of elution of studied lipid exosome extracts. See details in Figure S4.

### 2.2. Quantitative Densitometry for PL Classes

Results from HPTLC-UV densitometry show that an increase in PL, expressed as µg PL per 100 µg of exosome protein, were significant and gradually obtained when passing from fibroblast-derived (NIH-3T3) to moderate (B16-F1) and to highly metastatic (B16-F10)-derived exosomes (Table 2). Among the PL, the increase is due to SM, PC and PE. Contrarily, PS content is very low for all the studied samples.

On the other hand, UV at 190 nm only detects unsaturated lipids. In the case of SM, all structures, whether they contain unsaturated or saturated fatty acid chains, are detected at this wavelength as sphingosine moiety in their respective ceramides has a double bond. However, saturated fatty-acyl structures are not detected in the case of PC. The presence of saturated PC species was confirmed by HPTLC-ESI-MS. While the content in SM should be correct, the PC content would be underestimated by UV-densitometry since completely saturated PCs are not determined.

For this reason, primuline-induced fluorescence was used to quantify saturated PC. Primuline experiences increases in emission in the presence of long hydrocarbon chains, including saturated ones due to dipole-induced dipole interactions, as it was described elsewhere [20,21].

**Figure 5:**
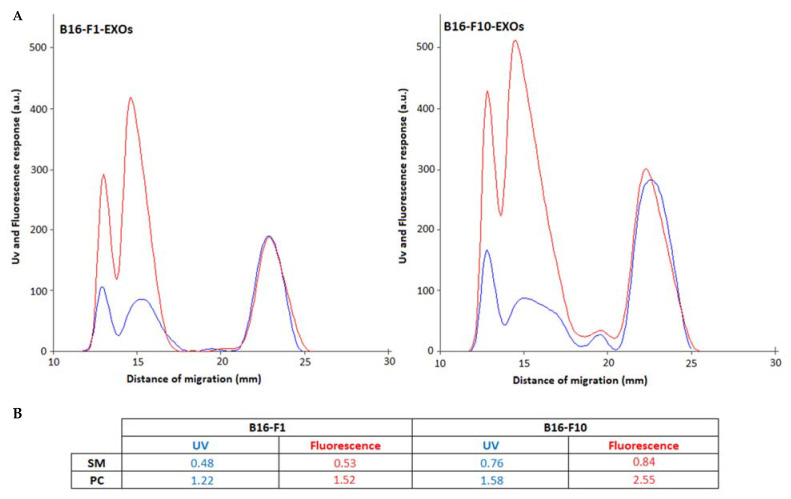
(**A**) UV-190 (blue) and primuline-induced fluorescence (red) densitograms corresponding to B16F1-EXOs and B16F10-EXOs. (see Experimental for impregnation and Fluorescence densitometry conditions). (**B**) Results from one-plate quantification of SM and PC classes in exosome lipid extracts by UV at 190 nm and primuline-induced fluorescence densitometry, using response factors of the corresponding standards, and expressed in µg of PL per 100 µg of exosome protein.

Figure 5 shows HPTLC-UV and primuline fluorescence chromatograms, and corresponding results on quantification of SM and PC for B16-F1-EXOs and B16-F10-EXOs, expressed in µg PL/100 µg exosome protein. As shown in this Figure, similar SM contents were found for B16-F1 and B16-F10 by both UV and primuline fluorescence densitometry. However, for PC, a higher content was found using primuline-induced fluorescence, due to the presence of saturated fatty acyl PC structures.

Primuline fluorescence densitometry confirms the result obtained by UV regarding the content of SM + PC (µg/100 µg protein) and the degree of tumor malignancy of the exosomes studied.

As a summary, this work describes the quantitative determination of exosomal PL classes by densitometry, and the ease of use, speed, reproducibility and selective access to a specific area of the plate, by using HPTLC-ESI-MS for identifying individual PL molecular species. This is of particular interest, because the interface is portable and the operation is complete within seconds

The results of HPTLC-UV densitometry (Table 2) show an agreement on the evolution of the content of SM + PC + PE when passing from fibroblasts to tumor cell lines in the studied samples. Significant variations in the concentration of PL have been determined with respect to the analytical uncertainties of the method, providing a repeatable analytical characterization in a reasonably short time. Differences on the aggressiveness of the cell line could be related to those results. However further studies should be performed.

## 3. Materials and Methods

### 3.1. Cell Culture Conditions

B16-F1 and B16-F10 cells, less and more metastatic murine skin melanoma cells respectively and NIH-3T3 cell (embryonic murine fibroblasts), were provided by cell services from Cancer Research-UK (London, UK). They were cultured in Dulbecco’s modified Eagle’s medium (DMEM) provided by Biowest (Nuaillé, France) supplemented with 10% of fetal bovine serum obtained from GIBCO laboratories (New York, NY, USA), 1% penicillin/streptomycin and 1% amphotericin provided by Biowest (Nuaillé, France) and maintained under normoxic conditions.

To obtain the culture media free of exosomes, they were depleted from serum by ultracentrifugation at 100,000× *g* for 8 h at 4 °C.

### 3.2. Exosome Isolation 

For exosomal purification, cells were seeded in cell culture plates until they reached confluence. Then, the cell culture medium was replaced by medium free of exosomes and cells were maintained during 48 h. NIH-3T3-EXOs, B16-F1-EXOs, B16-F10-EXOs and NIH-3T3-EXOs were purified by serial ultracentrifugation cycles from cell culture supernatants of parental cells until confluence. To remove remaining debris, supernatants were centrifuged for 20 min at 2000× *g* at 4 °C. Then, for the separation of the microvesicles, samples were centrifuged during 1 h at 10,000× *g* and at 4 °C. To isolate exosomes, the samples were ultracentrifuged twice for 2 h at 100,000× *g* and at 4 °C. The final precipitates were suspended in phosphate buffer saline and a Pierce BCA protein assay provided by Thermo Fisher Scientific (Waltham, MA, USA) was performed in order to estimate the protein content in the exosomal sample following the instructions provided by the manufacturer.

### 3.3. Total Lipid Isolation from Exosomes

Three biological replicates of exosomes from each cell line were collected and processed to minimize variation in lipid extraction and analysis between samples. Total lipid content of exosomes was extracted using a modified Folch extraction protocol [22].

Briefly, exosome suspensions were re-suspended in an ice cold chloroform (CHCl_3_: MeOH) (2:1, *v*/*v*) mixture. Samples were vortexed and mixed during 10 min at room temperature, and they were finally centrifuged at 6000× *g* for 10 min at room temperature. After that, a biphasic suspension separated by an interface containing proteins was formed. Bottom part (containing lipids in chloroform) was extracted using a glass pipette and was completely dried under an argon atmosphere and storage at −20 °C until use. 

### 3.4. Standards and Chemicals

N-Hexadecanoyl-D-erythro-sphingosylphosphorylcholine from chicken egg (SM d18:1/16:0; >99%; 383907-87-7 CAS), 1,2-dioleoyl-*sn*-glycero-3-phosphoethanolamine (PE 18:1/18:1, Δ9-Cis DOPE; >99%; 4004-05-1 CAS), 1,2-dioleoyl-*sn*-glycero-3-phospho-L-serine (sodium salt, PS 18:1/18:1, DOPS; >99%, 90693-88-2 CAS), 1-palmitoyl-2-oleoyl-*sn*-glycero-3-phosphoinositol (ammonium salt, PI 16:0/18:1; >99%; 50730-13-7 CAS), 1-palmitoyl-2-oleoyl-*sn*-glycero-3-phosphate (sodium salt, PA 16:0/18:1; >99%; 169437-35-8 CAS), D-glucosyl-*β*-1,1′-N-palmitoyl-D-erithro-sphingosine, (GlcCer d18:1/16:0; >99%; 74365-77-8 CAS), were obtained from Avanti Polar Lipids (Alabaster, AL, USA).

L-*α*-phosphatidylcholine (from egg yolk, Type XVI-E; PC; ≥99%; 8002-43-5 CAS), and L-*α*-phosphatidyl-DL-glycerol (from egg yolk, PG; ≥99%; 80146-86-7 CAS) were purchased from Sigma-Aldrich (Madrid, Spain). Cholesterol, (CHOL; ≥90%, 57-88-5 CAS) was from EMD Millipore Corp. (Billerica, MA, USA). 

Standards were used to optimize the separation of the targeted PL from other exosome lipids, for setting up calibration curves, and as reference for MS analysis.

For a given PL class, the notation adopted for the identity of a molecular species was (x:y) where x is the carbon number of the fatty acid-chains of the molecule; and y corresponds to the total number of double bonds.

Methanol (MeOH, HPLC-grade, 99.9%), dichloromethane (DCM, HPLC-grade, 99.5%), and tetrahydrofuran (THF, HPLC-grade, 99.5%) were purchased from Scharlab (Barcelona, Spain). Chloroform (CHCl_3,_ HPLC-grade, 99.0%), acetone (HPLC grade, 99%), and acetic acid glacial (AcH, 99.5%) were purchased from Panreac (Barcelona, Spain).

HPTLC silica gel 60 plates (20 × 10 cm) from Merck (Darmstadt, Germany) were employed. They were pre-washed with THF and kept in desiccator in N_2_ atmosphere.

Primuline (dye content 50%; 8064-60-6 CAS) was purchased from Sigma-Aldrich (Madrid, Spain).

### 3.5. HPTLC-Densitometry

Instruments for sample application, chromatographic development, densitometry, plate impregnation and HPTLC-MS coupling were purchased from CAMAG (Muttenz, Switzerland).

Samples from exosome lipid extracts were dissolved in DCM:MeOH, 1:1 *v*/*v*, in order to obtain a concentration of 10 mg/mL of exosomal protein in each sample. 25 μL/band were applied on the corresponding HPTLC silica gel plate in triplicate (250 μg of exosomal protein/band), as 4-mm bands, by using the Automatic TLC Sampler (ATS4) system. Solutions of each above individual standards were also applied in triplicate on the same plate (concentration: 1 mg/ml per standard in DCM:MeOH, 1:1 *v*/*v*; application volume: 1 μl/band). In order to optimize the applied sample volume and thus save sample, the ATS4 filling quality method was used. In a given plate, minimal distance between tracks was 6 mm; distances from the lateral and lower plate edges were 10 mm. One or more tracks were left empty, as blanks. 

Isocratic chromatographic development up to 30 mm-migration distance was performed in a horizontal developing chamber (20 × 10 cm) using a mixture of CHCl_3_, MeOH, acetone, water, AcH (6:2:8:1:2, *v*/*v*/*v*/*v*/*v*) for separating N-containing phospholipid (PL) classes between them and from all other lipids in lipid exosome extracts. 

Three plates per lipid extract sample were developed on different days. 

Detection was first carried out using a TLC Scanner 3 densitometer in mode UV at 190 nm. Then, one of these plates was submitted to post-impregnation by dipping it into a solution of primuline in MeOH (200 mg L^−1^) using a chromatogram Immersion Device III. Induced-fluorescence was collected by densitometry in fluorescence mode, at λ_em_ > 400 nm after excitation at λ_exc_ = 365 nm.

Baseline of chromatograms was corrected manually. WinCATS software (v 1.4.3.6336) from CAMAG (Muttenz, Switzerland) was used to control and process data from sample application, chromatography and densitometry.

Intra- and inter-plate repeatability (as RSD% and Area interval for 95% confidence interval) were calculated for standard and samples (n = 3 × 3). For the same cell line, lipid extracts from different exosome extraction operations led to repeatable chromatograms.

Mass (μg) of each PL class was calculated from the corresponding standard response factor, and expressed per 100 μg of the corresponding total exosome protein.

### 3.6. HPTLC-MS

Non-impregnated plates were utilized for MS coupling through the TLC-MS Interface 2, equipped with an oval, 4 × 2-mm extraction head which was positioned on the corresponding PL-band maximum, whose the X,Y coordinates were provided by WinCats software, using a laser crosshair.

Then the interface head was lowered. MeOH was delivered for band extraction at 0.2 mL/min by using a PU-2080 HPLC pump (Jasco, Tokyo, Japan). The eluate is directed through a 2-μm stainless steel frit to remove silica gel and then sent to the mass spectrometer. Electrospray (ESI)-MS was conducted in positive (ESI^+^) and negative (ESI^−^) modes, and mass spectra (MS) were registered on an Ion trap Amazon Speed Spectrometer (Bruker Daltonics, Bremen, Germany).

For each separated PL class on the plate, three ESI-MS experiments were done from band replicates. First, HPTLC-ESI-MS spectra were performed in both (ESI^+^) and (ESI^−^) modes. Once the ionization mode was selected, HPTLC-ESI-MS/MS was used for identity confirmation. These experiments were performed from different plates.

Bruker Daltonics Trap Control software packages v 8.0 and Data Analysis v 5.2 were used to control the mass spectrometer and process data.

HPTLC-ESI-MS/MS operating conditions are specified for each case in the Results and Discussion section or Supplementary Information. 

For lipid identification, LIPID MAPS software was used (http://www.lipidmaps.org).

For spectra interpretation, formation of sodiated background clusters [Na^+^(CH_3_-COONa)_n1_, (n1, n2 ≥ 0), with *m*/*z* Δ = 82 for sodium acetate, and sample ion clusters [M+Na]^+^(CH_3_-COONa), were considered as artifacts [23,24].

## Figures and Tables

**Table 1:** Differential characteristics of HPTLC-densitometry-ESI.MS.

**Inherent characteristics of HPTLC-densitometry-MS**
- Standards and many samples run in parallel on the same chromatographic plate - As there is no column, no retention of substances onto the column occurs. - All sample components are on the plate and can be detected by UV or FL - Removal of mobile phase after HPTLC step and recovery of separated bands using a single solvent. Similar ionization for all the lipids belonging to a given class. - Speed of access to the selected hot bands on the plate using the described HPTLC-MS coupling system - Precision and representativeness of band extraction
**Differential characteristics of HPTLC vs. shotgun MS by direct infusion**
- In shotgun there is no previous separation. This technique usually presents ion suppression effects, which are minimized through prior chromatographic separation, as in HPTLC. - Likewise, in shotgun there are peak overlaps due to the presence of isomeric and/or isobaric lipids.
**Differential characteristics vs. HPTLC on LC-MS**
LC-MS is more efficient in separation than HPTLC. LC-MS quantification is complicated as there are variations in ionization of molecules of the same lipid class due to differences in the chemical environment ionization at different retention times. HPTLC quantification is usually carried out by densitometry. No studies have been published on HPTLC-MS quantification

**Table 2:** Results from quantification of PL classes in exosome lipid extracts by UV densitometry at 190 nm, using response factors of the corresponding standards (n = 3 × 3), and expressed in µg of PL per 100 µg of protein, as detailed in Experimental and in Figure S2. Average of three plates. For quantification and for each PL class, the corresponding standard was previously selected on the basis of the identity of the most intense ion.

PL	NIH-3T3-Exos	B16-F1-Exos	B16-F10-Exos
SM	0.50 (RSD: 18.00%)	0.48 (RSD: 17.40%)	0.76 (RSD: 8.50%)
PC	0.79 (RSD: 15.80%)	1.22 (RSD: 6.30%)	1.58 (RSD: 2.60%)
PS	0.08 (RSD: 11.80%)	0.02 (RSD: 55.00%)	0.08 (RSD: 47.60%)
PE	0.20 (RSD: 8.00%)	1.99 (RSD: 2.80%)	3.70 (RSD: 1.60%)

## Supplementary Materials

The following are available online at https://www.mdpi.com/article/10.3390/ijms23031150/s1.
