# Supplementary material for: High-Performance Thin-Layer Chromatography-Densitometry-Tandem ESI-MS to Evaluate Phospholipid Content in Exosomes of Cancer Cells
Source: Int J Mol Sci. 2022 Jan 21;23(3):1150. doi: 10.3390/ijms23031150 (PMC8835402; doi:10.3390/ijms23031150)

Supporting information

High-Performance Thin-Layer Chromatography-Densitometry-Tandem ESI-MS to evaluate phospholipid content in exosomes of cancer cells

María Sancho-Albero[1,2,+,*], Carmen Jarne[3,x,*], María Savirón[4], Pilar Martín-Duque[2,5,6,#], Luis Membrado[3], Vicente L. Cebolla[3,*], Jesús Santamaría[1,2]

[1] Department of Chemical Engineering, Aragon Institute of Nanoscience (INA), University of Zaragoza, Zaragoza. Spain
[2] Networking Research Center on Bioengineering Biomaterials and Nanomedicine (CIBER-BBN), Madrid, Spain
[3] Instituto de Carboquímica, ICB-CSIC, Zaragoza, Spain
[4] CEQMA-CSIC, Facultad de Ciencias, Universidad de Zaragoza, Spain
[5] Instituto Aragonés de Ciencias de la Salud// IIS Aragón, Zaragoza, Spain
[6] Fundación Araid, Zaragoza, Spain
[+] Present Address: Department of Molecular Biochemistry and Pharmacology, Instituto di Richerche Farmachologiche Mario Negri IRCCS, 20156, Milano, Italy
[x] Present Address: Institute of Catalysis and Petrochemistry, ICP-CSIC, Madrid, Spain
[#] Present Address: Surgery Department. School of Medicine, University of Zaragoza, Zaragoza. Spain
* Correspondence: Vicente L. Cebolla: vcebollla@icb.csic.es; (+34)976733977, Carmen Jarne: c.jarne@csic.es; (+34) 915854800, and María Sancho-Albero: msancho@unizar.es; (+34)695347470

Figure S1.- HPTLC chromatographic development of standards and front of elution (m.d.≥ 30 mm). Structure of standards *(see Materials and Methods):* ***1****, SM;* ***2, PC;*** ***3****, PS;* ***4****, PI;* ***5****, PE;* ***6****, PA;* ***7****, HexCer;* ***8****, Chol*

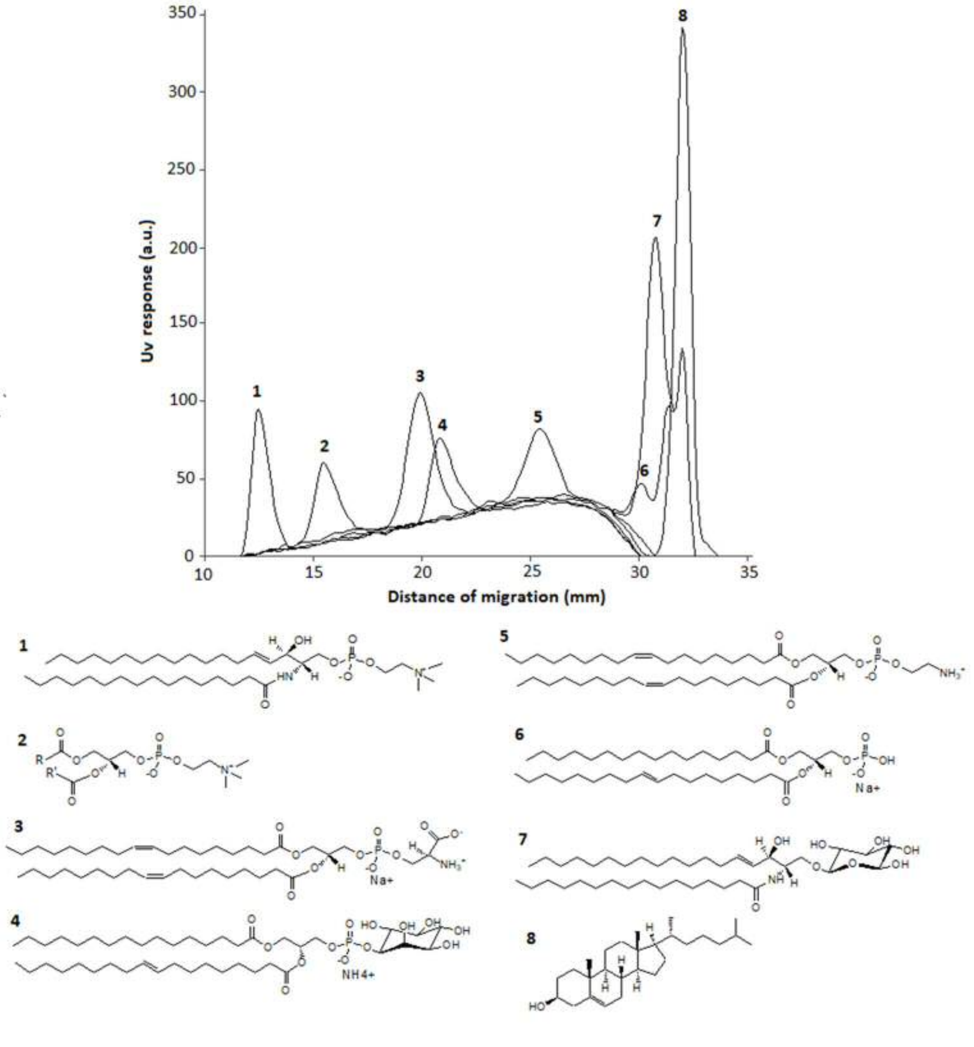

Figure S2.- Intra- and inter-plate HPTLC repeatability results for the standards (A) and PL classes of the B16-F1-EXOs (B), B16-F10-EXOs (C), and NIH-3T3-EXOs (D) including the RSD%, ± Area (for a 95% confidence interval, CI).

A

	Intra-plate Repeatability						Inter-plate Repeatability		
	Plate 1		Plate 2		Plate 3				
	Average peak area	%RSD	Middle pic Area	%RSD	Average peak area	%RSD	Average area	%RSD	CI (95%)
SM	1071.3	2.5	1067.5	2.5	1111.0	5.6	1083.2	3.9	289.1
PC	735.4	0.4	745.8	1.0	803.3	4.3	761.5	4.8	251.4
PS	1406.2	2.9	1297.2	1.0	1208.6	3.2	1304.0	6.9	626.3
PE	1039.2	0.1	992.4	7.9	835.6	2.4	955.7	10.5	697.2

B

	Inter-plate Repeatability				
	Peak area Plate 1	Peak area Plate 2	Peak area Plate 3	Average area	%RSD
SM	1173.0	1088.9	828.8	1030.2	17.4
PC	1931.1	1923.6	1723.1	1859.3	6.3
PS	15.9	50.0	59.8	41.9	55.0
PE	3857.7	3686.4	3884.3	3809.5	2.8

C

	Inter-plate Repeatability				
	Peak area Plate 1	Peak area Plate 2	Peak area Plate 3	Average area	%RSD
SM	1736.6	1716.1	1485.9	1646.2	8.5
PC	2382.5	2484.9	2367.7	2411.7	2.6
PS	98.3	224.4	289.1	203.9	47.6
PE	7112.8	6940.1	7151.8	7068.2	1.6

D

	Intra-plate Repeatability	
	Average peak area Plate 1	%RSD
SM	1089.1	18.0
PC	1205.7	15.8
PS	206.5	11.8
PE	382.9	8.0

Figure S3.- HPTLC-ESI-MS spectra of standards

S3-1.- SM, PC, PS and PE standards

A) HPTLC-ESI^{+}-MS spectrum of SM standard
B) HPTLC-ESI^{+}-MS/MS spectrum of ion precursor at *m*/*z* 725.6 (SM standard)
C) HPTLC-ESI^{+}-MS spectrum of PC standard
D) HPTLC-ESI^{+}-MS/MS spectrum of ion precursor at *m*/*z* 782.6 (PC standard)
E) HPTLC-ESI^{-}-MS spectrum of PC standard
F) HPTLC-ESI^{-}-MS spectrum of PS standard
G) HPTLC-ESI^{-}-MS/MS spectrum of ion precursor at *m*/*z* 786.8 (PS standard)
H) HPTLC-ESI^{-}-MS spectrum of PE standard
I) HPTLC-ESI^{-}-MS/MS spectrum of ion precursor at *m*/*z* 742.8 (PE standard)
J) HPTLC-ESI^{+}-MS spectrum of PE standard

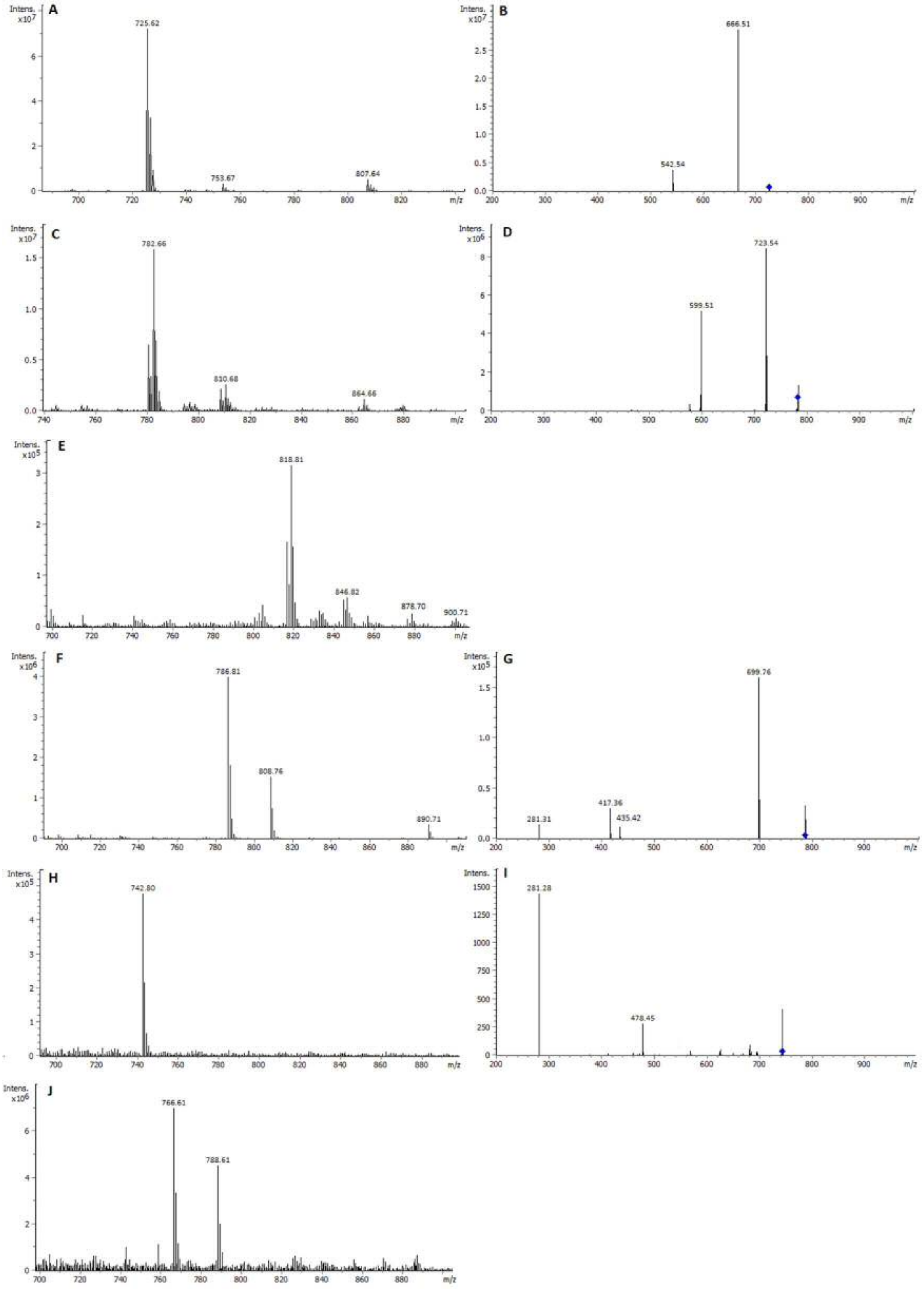

S3-2.- phosphatidylinositol (PI), phosphatidic acid (PA) and glucosyl ceramide (GlcCer) standards

A) HPTLC-ESI^{-}-MS spectrum of PI standard

B) HPTLC-ESI^{-}-MS/MS spectrum of ion precursor at *m/z* 835.8 (PI standard)

C) HPTLC-ESI^{-}-MS spectrum of PA standard

D) HPTLC- ESI^{-}-MS/MS spectrum of ion precursor at *m/z* 673.9 (PA standard)

E) HPTLC-ESI^{+}-MS spectrum of GlcCer standard

F) HPTLC-ESI^{+}-MS/MS spectrum of ion precursor at *m/z* 722.6 (GlcCer standard)

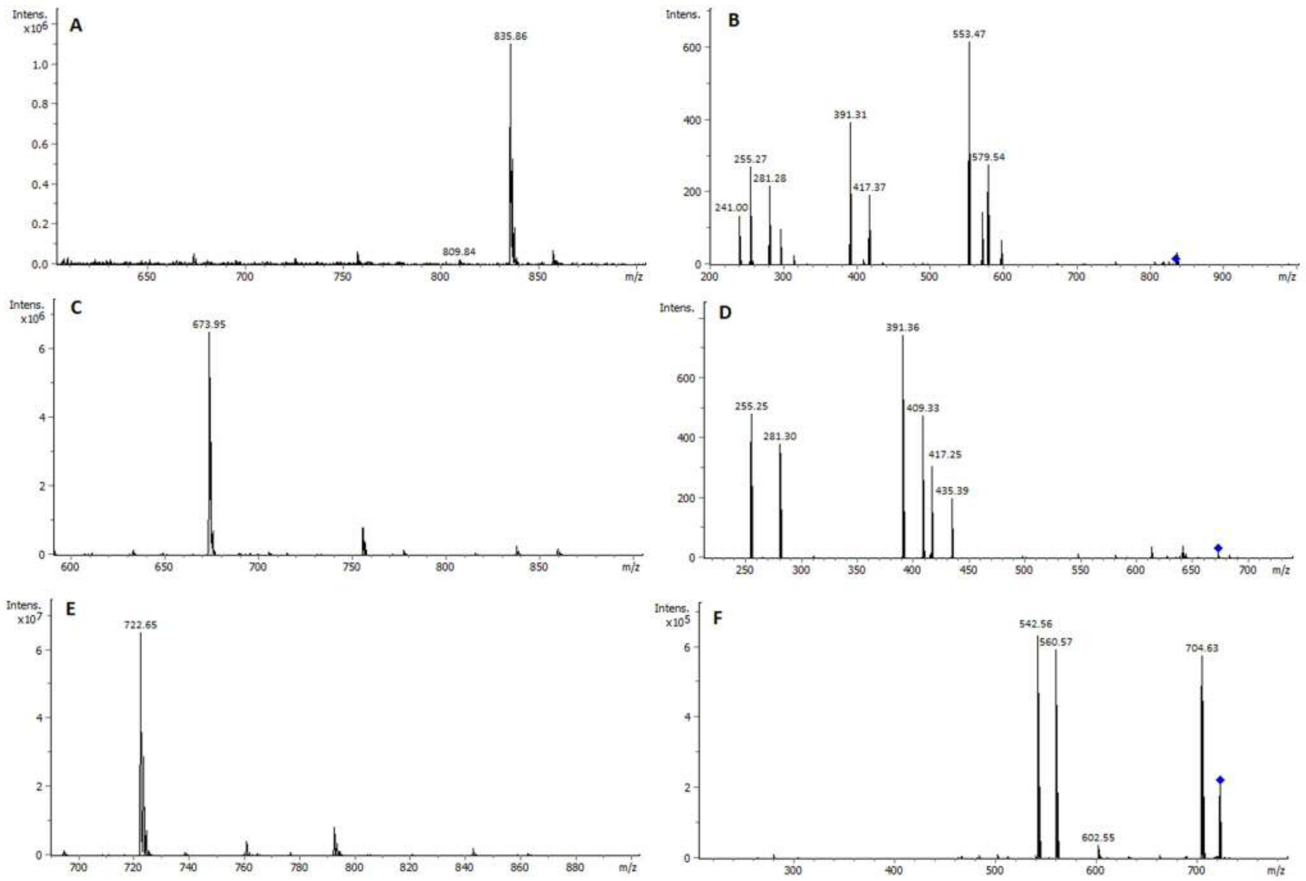

Figure S4.-HPTLC-ESI-MS spectra of SM, PC, PS and PI from NIH-3T3-EXOs, B16-F1-EXOs and B16-F10-EXOs.

S4-1.- Example of repeatability of SM from different exosome batches (1 and 2)

A) HPTLC-ESI^{+}-MS spectrum of B16-F1-EXOs (batch 1; peak at 14.6 mm-m.d.)

B) HPTLC-ESI^{+}-MS spectrum of B16-F1-EXOs (batch 2; peak at 14.6 mm-m.d.)

C) HPTLC-ESI^{+}-MS spectrum of B16-F10-EXOs (batch 1; peak at 14.5 mm-m.d.)

D) HPTLC-ESI^{+}-MS spectrum of B16-F10-EXOs (batch 2; peak at 14.5 mm-m.d.)

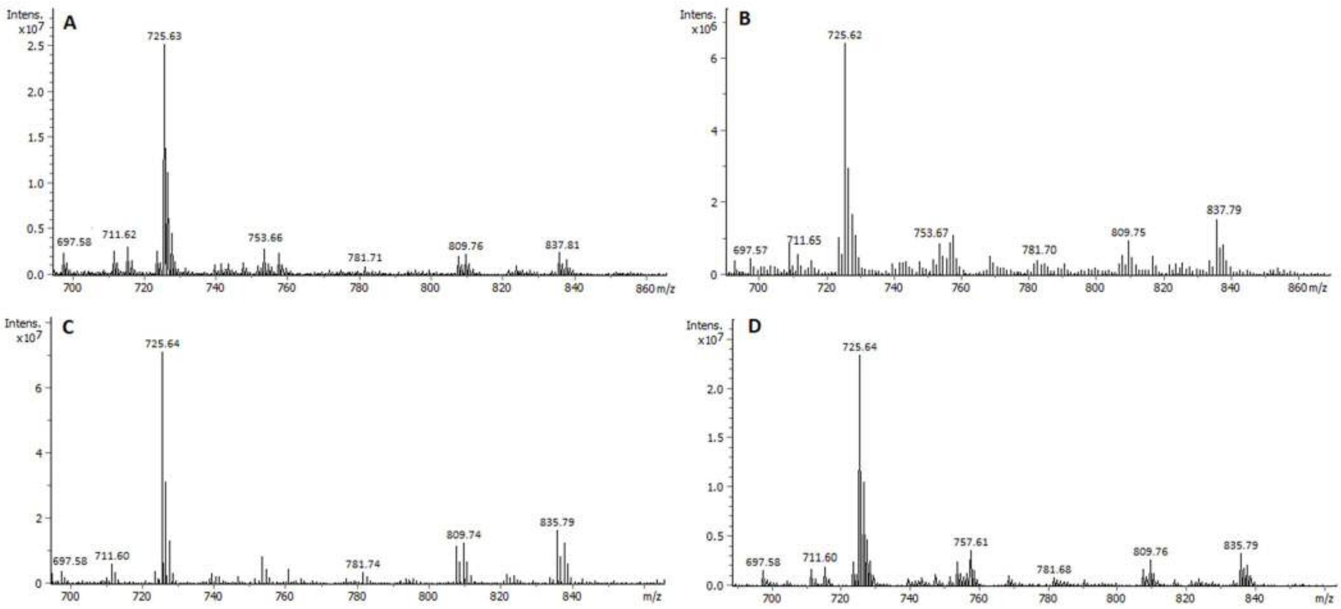

S4-2.- Sphingomyelins (SM)

A) HPTLC-ESI+-MS spectrum of NIH-3T3-EXOs (peak at 14.0 mm-m.d.)

B) HPTLC-ESI+-MS/MS spectrum of ion precursor at *m/z* 725.6 (NIH-3T3-EXOs)

C) HPTLC-ESI+-MS spectrum of B16-F1-EXOs (peak at 14.6 mm-m.d.)

D) HPTLC-ESI+-MS/MS spectrum of ion precursor at *m/z* 725.6 (B16-F1-EXOs)

E) HPTLC-ESI--MS spectrum of B16-F1-EXOs (peak at 14.6 mm-m.d.)

F) HPTLC-ESI+-MS spectrum of B16-F10-EXOs (peak at 14.5 mm-m.d.)

G) HPTLC-ESI+-MS/MS spectrum of ion precursor at *m/z* 725.6 (B16-F10-EXOs)

H) HPTLC-ESI--MS spectrum of B16-F10-EXOs (peak at 14.5 mm-m.d.)

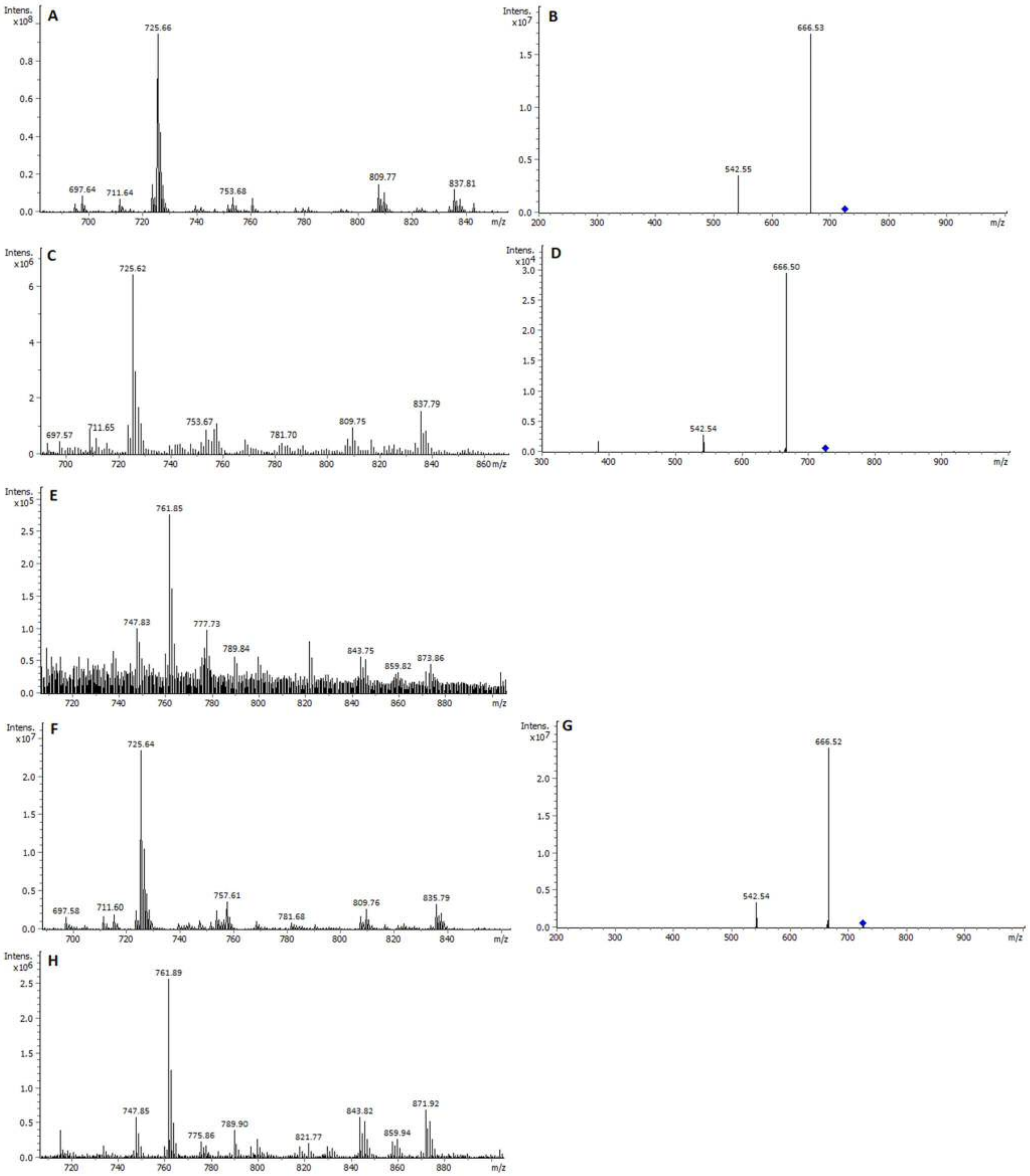

S4-3.- Phosphatidylcholines (PC)

A) HPTLC-ESI$^+$-MS spectrum of NIH-3T3-EXOs (peak at 19.1 mm-m.d.)

B) HPTLC-ESI$^+$-MS/MS spectrum of precursor ion at *m/z* 768.7 (NIH-3T3-EXOs)

C) HPTLC-ESI$^+$-MS spectrum of B16-F1-EXOs (peak at 20.3 mm-m.d.)

D) HPTLC-ESI$^+$-MS/MS spectrum of precursor ion at *m/z* 782.6 (B16-F1-EXOs)

E) HPTLC-ESI$^-$-MS spectrum of B16-F1-EXOs (peak at 20.3 mm-m.d.)

F) HPTLC-ESI$^+$-MS spectrum of B16-F10-EXOs (peak at 18.0 mm-m.d.)

G) HPTLC-ESI$^+$-MS/MS spectrum of precursor ion at *m/z* 782.6 (B16-F10-EXOs)

H) HPTLC-ESI$^-$-MS spectrum of B16-F10-EXOs (peak at 18.0 mm-m.d.)

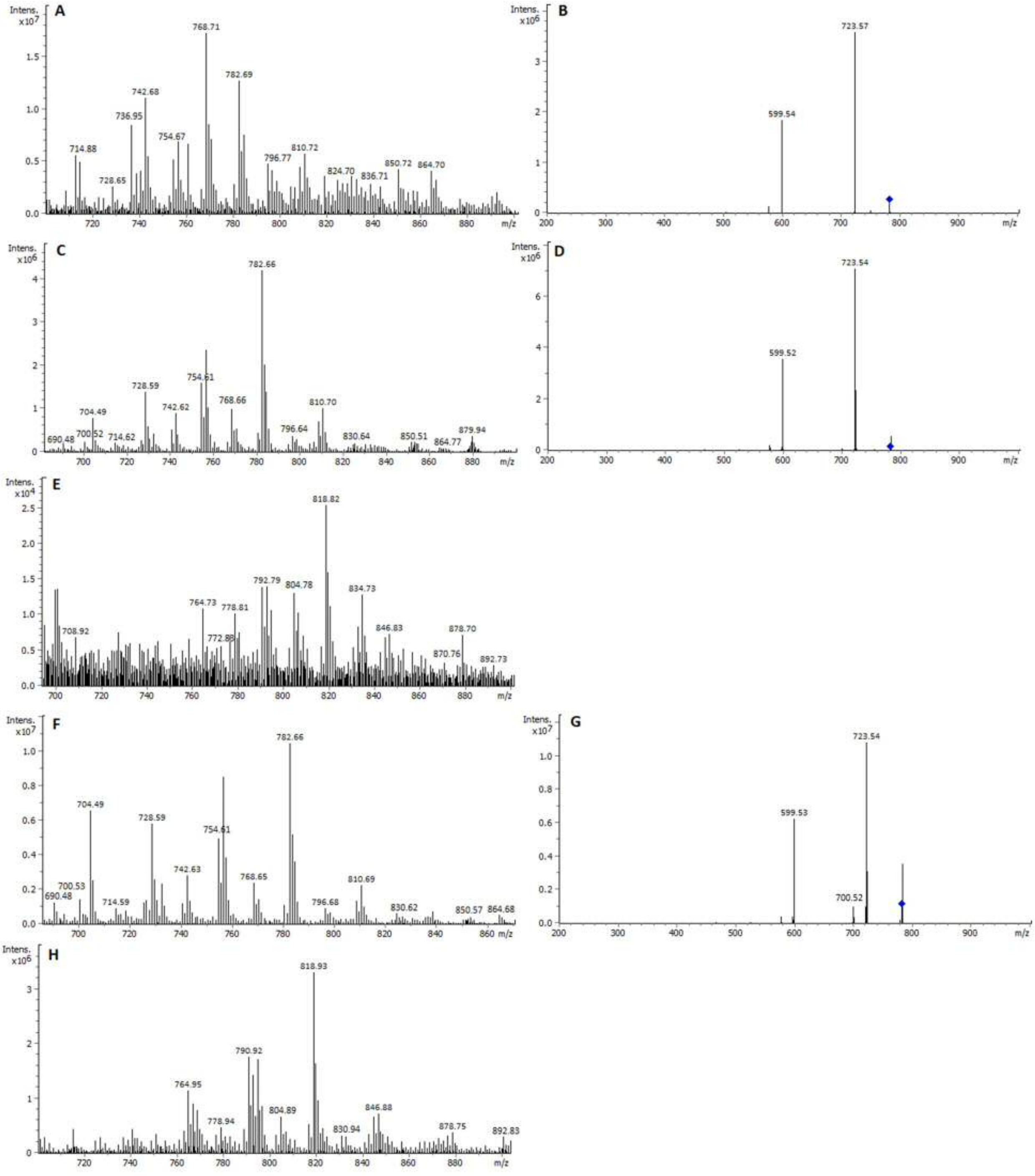

S4-4.- Phosphatidylserines (PS)

A) HPTLC-ESI$^-$-MS spectrum of B16-F1-EXOs (peak at 23.8 mm-m.d.)

B) HPTLC- ESI$^-$-MS/MS spectrum of precursor ion at 786.7 *m/z* (B16-F1-EXOs)

C) HPTLC-ESI$^-$-MS spectrum of B16-F10-EXOs (peak at 22.6 mm-m.d.)

D) HPTLC- ESI$^-$-MS/MS spectrum of precursor ion at *m/z* 786.8 (B16-F10-EXOs)

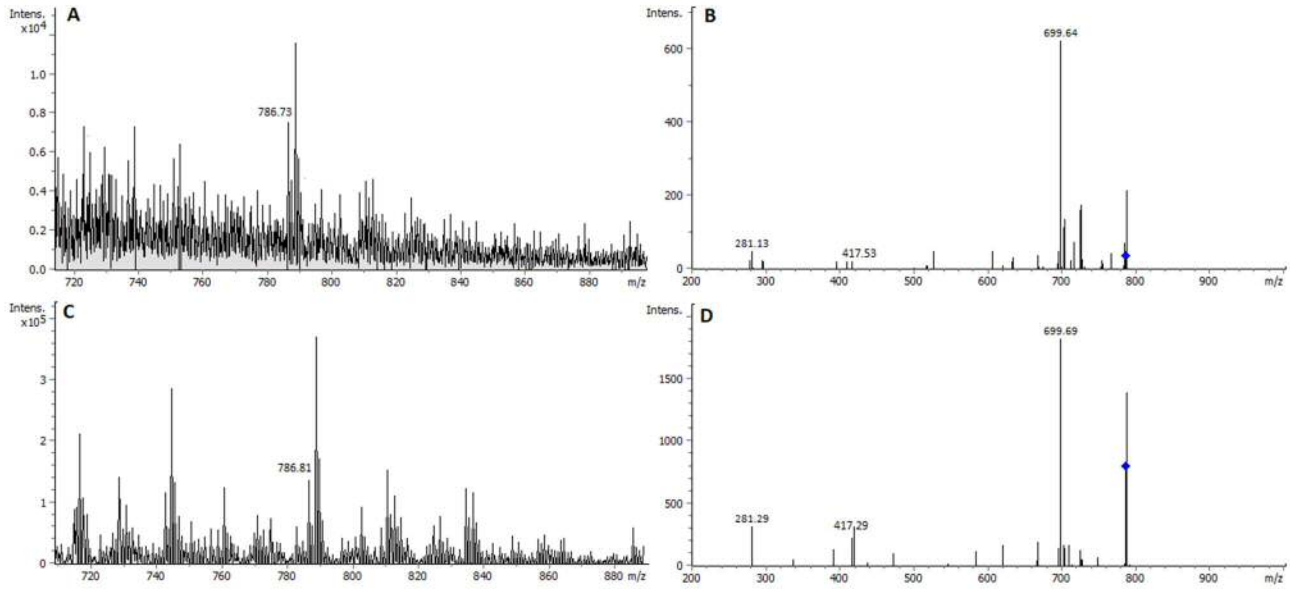

S4-5.- Phosphatidylethanolamines (PE)

A) HPTLC-ESI$^-$-MS spectrum of NIH-3T3-EXOs (peak at 24.8 mm-m.d.)
B) HPTLC- ESI$^-$-MS/MS spectrum of precursor ion at *m/z* 742.9 (NIH-3T3-EXOs)
C) HPTLC-ESI$^-$-MS spectrum of B16-F1-EXOs (peak at 25.9 mm-m.d.)
D) HPTLC- ESI$^-$-MS/MS spectrum of precursor ion at *m/z* 742.7(B16-F1-EXOs)
E) HPTLC-ESI$^+$-MS spectrum of B16-F1-EXOs (peak at 25.9 mm-m.d.)
F) HPTLC-ESI$^-$-MS spectrum of B16-F10-EXOs (peak at 22.6 mm-m.d.)
G) HPTLC- ESI$^-$-MS/MS spectrum of precursor ion at *m/z* 742.8 (B16-F10-EXOs)
H) HPTLC-ESI$^+$-MS spectrum of B16-F10-EXOs (peak at 22.6 mm-m.d.)

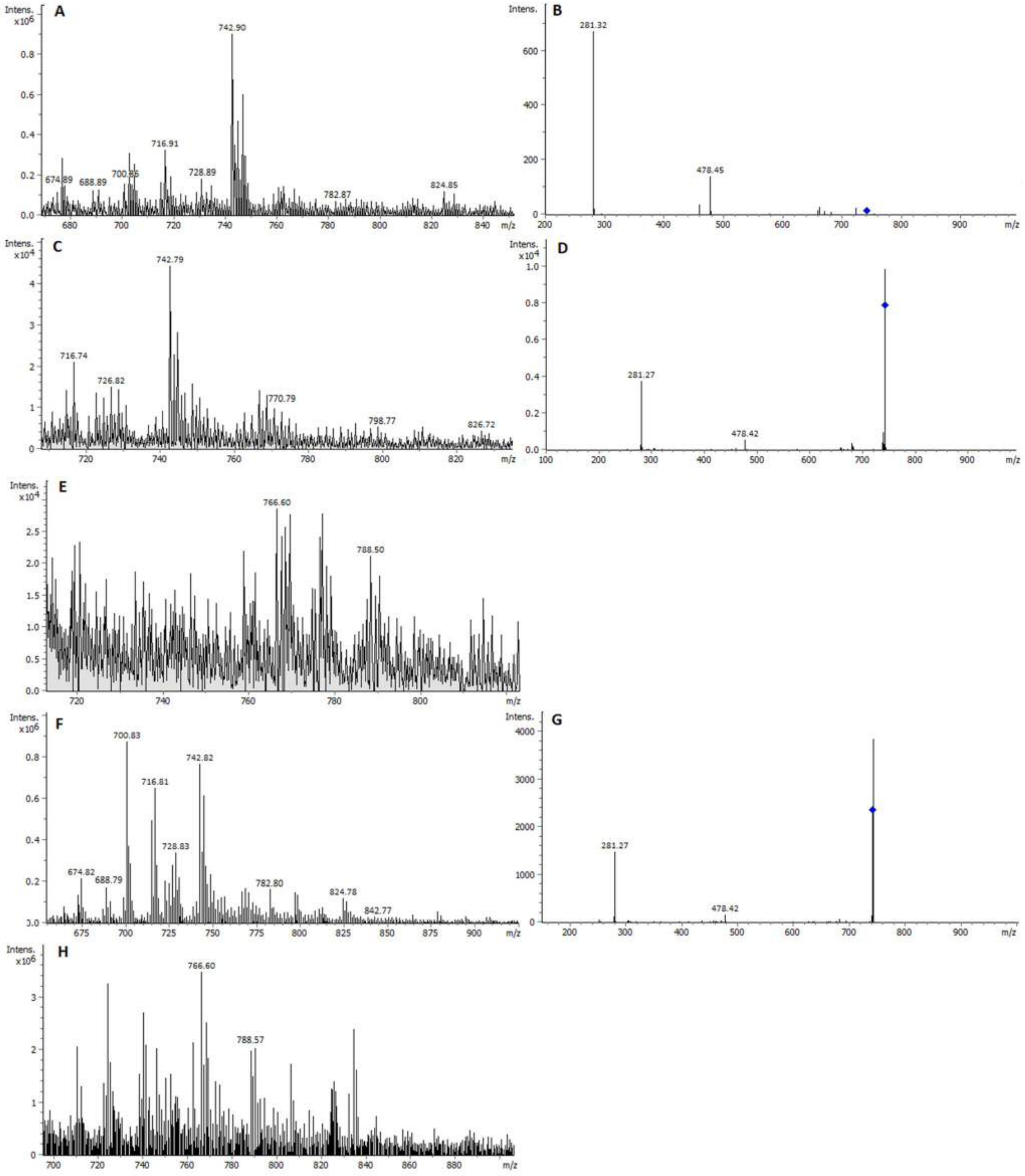

S4-6.- Front of elution (band at 30 mm-m.d.)

A) HPTLC-ESI^{+}-MS spectrum of B16-F1-EXOs

B) HPTLC-ESI^{-}-MS spectrum of B16-F1-EXOs

C) HPTLC-ESI^{+}-MS spectrum of B16-F10-EXOs

D) HPTLC-ESI^{-}-MS spectrum of B16-F10-EXOs

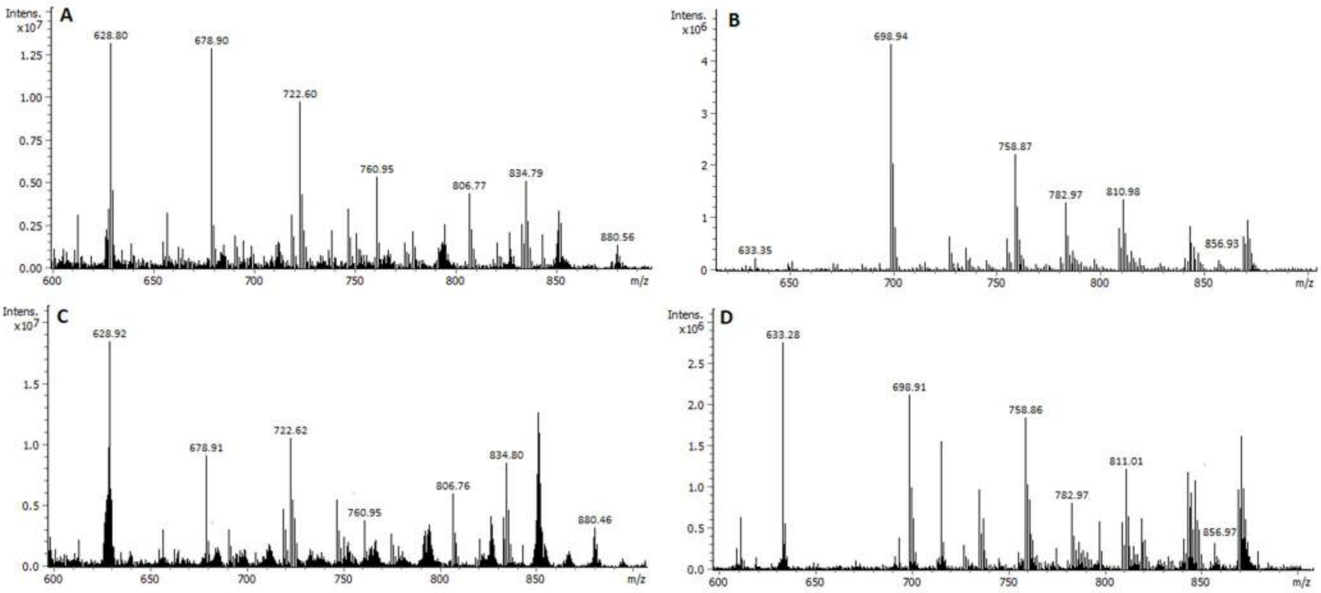

Supplement: Supplementary file 1 [file ijms-23-01150-s001.zip › ijms-1537132-supplementary.pdf]
